# Clinical Use of Valproate and Divalproex in Autism Spectrum Disorder: A Systematic Review of Efficacy and Safety

**DOI:** 10.7759/cureus.114420

**Published:** 2026-08-12

**Authors:** FNU Hridhey, Nihit Gupta, Priyal Khurana, Victoria Miller, Mayank Gupta

**Affiliations:** 1 Psychiatry and Behavioral Sciences, Epionique Mental Healthcare, Summersville, USA; 2 Psychiatry, Dayton Children's Hospital, Dayton, USA; 3 Clinical Psychology, Mahatma Jyoti Rao Phoole University, Jaipur, IND; 4 Psychiatry, Touro College of Osteopathic Medicine, New York, USA; 5 Psychiatry and Behavioral Sciences, Loma Linda University School of Medicine, Loma Linda, USA

**Keywords:** aggression, a systematic review, autism spectrum disorder (asd), pervasive developmental disorder, valproate

## Abstract

The aim of this systematic review is to appraise the current evidence on the efficacy and safety of valproate (VPA), including divalproex sodium (DVPX), in managing irritability, aggression, repetitive behaviors, and other associated symptoms, mostly in the pediatric population with autism spectrum disorder (ASD). Major medical literature databases were searched for randomized controlled trials (RCTs), open-label trials, and any other relevant studies or clinical trials reporting on patients with ASD (predominantly children and adolescents) treated with DVPX/VPA for any reason. A total of 654 abstracts were screened, and four clinical trials were selected for inclusion. Out of these four clinical trials, three were RCTs (n = 13, 27, and 30), and one was an open-label trial. Data were extracted using a standardized form focused on study design and validity domains. Two reviewers independently abstracted data, with discrepancies resolved by consensus. Meta-analysis was not performed due to a lack of homogeneity among the three RCTs. Although most of the studies reported improved overall symptoms, they had different outcome measures. The quality of evidence available is low, and there is a need for higher-powered studies in the future. There is insufficient evidence to make conclusive recommendations on DVPX/VPA for improvement in core symptoms in the pediatric population. However, DVPX/VPA may represent a potential off-label option for selected patients with significant irritability, aggression, and repetitive behaviors associated with ASD.

## Introduction and background

Pharmacotherapy for autism spectrum disorder (ASD) has narrow evidence-based options. Risperidone, approved in 2006, and aripiprazole, approved in 2009, remain the only U.S. Food and Drug Administration (FDA)-approved agents indicated for irritability associated with ASD rather than for the core deficits [1]. These limited therapeutics do not match clinical needs, given ASD prevalence is now estimated at one in 31 children by age eight in the United States [2]. Because most affected children carry at least one co-occurring psychiatric or medical condition, clinicians routinely manage epilepsy, mood instability, aggression, and disruptive behaviors [3]. As a result, most medications in ASD, including divalproex sodium/valproate (DVPX/VPA), are prescribed off-label. These practices are common and often necessary but based on weaker evidence [4].

Antipsychotics are effective for irritability but carry a risk of metabolic syndrome, weight gain, hyperprolactinemia, and extrapyramidal symptoms that limit their tolerability, particularly with long-term use [1]. For this reason, clinicians continue to look for alternative treatments. DVPX/VPA is often considered because it is cost-effective, familiar to most prescribers, available in weight-based and serum-level-guided dosing, and widely used for pediatric mood instability and seizure disorders that frequently co-occur with ASD. The clinical evidence, however, needs to be clear, as DVPX/VPA was not developed for ASD. Its use in this population is calculated from its effectiveness as an anticonvulsant and mood stabilizer in other conditions.

DVPX is a stable compound made by combining valproic acid and sodium valproate, whereas VPA refers to the active form of the drug. Its formulation reduces stomach irritation and improves gastrointestinal tolerability compared with valproic acid. After absorption, both release the same active VPA in the body and produce the same therapeutic effects. DVPX/VPA actions are diverse, and several converge on circuits associated with ASD. The clearest effect is on gamma-aminobutyric acid (GABA), which raises GABA levels by blocking two enzymes (GABA transaminases and succinic semialdehyde dehydrogenase) and by increasing the activity of the enzyme that makes it (glutamic acid decarboxylase). The effect is a shift from excitatory glutamate signaling toward inhibition [5,6]. It also blocks voltage-gated sodium channels and T-type calcium channels, dampening the high-frequency, repetitive neuronal firing associated with both seizures and limbic hyperexcitability [6]. Because disruption of the excitatory/inhibitory balance in cortical and limbic circuits is one of the more consistent findings in the ASD pathophysiology empirical literature, an agent that shifts this balance toward inhibition offers a reasonable pathway to reduced behavioral reactivity, independent of any effect on core developmental symptoms [7].

A second mechanism operates at the level of gene expression (considered for its effects on mood for bipolar disorder). VPA is a broad-spectrum inhibitor of class I and class IIb histone deacetylases (HDACs), producing hyperacetylation of histones H3 and H4 and altering transcription of genes involved in neuroplasticity, cell survival, and synaptic remodeling [5,8]. Postnatally, in an already-formed nervous system, the same mechanism is hypothesized to support neuroplastic and mood-stabilizing effects, but this remains a reasonable inference rather than an ASD-specific finding.

A third effect is less well known, VPA may also decrease neuroinflammation. Valproate's HDAC-inhibiting properties lead to suppression of nuclear factor kappa B (NF-κB) signaling and downregulation of pro-inflammatory microglial activation in preclinical models. Most of the evidence comes from neuroprotection research on dopaminergic neurons [9]. Given the growing interest in immune dysregulation as a contributor to behavioral symptoms in some individuals with ASD, this anti-inflammatory property is a plausible mechanism by which VPA could influence behavior. However, this claim remains hypothetical, as no ASD treatment trial has tested it.

When related to symptoms seen in ASD, these mechanisms provide a possible, although not yet proven, explanation. Increased GABA activity and reduced brain excitability may help reduce irritability, aggression, and emotional reactivity. Effects on neuroplasticity and mood regulation through HDAC inhibition may improve mood instability and bipolar-like symptoms. In addition, the drug's effects on sodium and calcium channels may be particularly helpful for individuals with ASD who also have epilepsy or abnormal electroencephalogram (EEG) findings. None of these mechanisms predicts an effect on the core social-communication or restricted/repetitive symptom domains, and the clinical evidence for other symptoms also needs empirical validation and critical appraisal of the studies on these clinical phenotypes. 

Caution is warranted given histone deacetylase inhibition and disruption of excitatory/inhibitory synaptic balance that make DVPX/VPA reasonable after birth, and these are also the leading explanations for why prenatal exposure to VPA is linked to ASD features in rodent and primate studies [10]. This is not a contradiction so much as a reminder that valproate's effects are stage-dependent: the developing nervous system and the mature one respond differently to the same epigenetic alteration. This means that any discussion on the DVPX/VPA effect in ASD has to sit alongside its reproductive risks, which we address in this review.

Despite its common off-label use, evidence for valproate in ASD is still limited, with studies showing differences in design and inconsistent results. No previous review has organized the evidence according to the clinical symptoms for which valproate is commonly used. Therefore, we reviewed the available literature on valproate in ASD, evaluated eligible clinical trials, and organized the findings based on the clinical symptoms for which valproate is considered in practice.

## Review

Methods

Our systematic review was performed according to the Preferred Reporting Items for Systematic Review and Meta-Analysis (PRISMA) guidelines. The protocol was registered at PROSPERO: CRD420261448132, and no deviations from the registered protocol occurred.

Data sources searched

**Figure 1:**
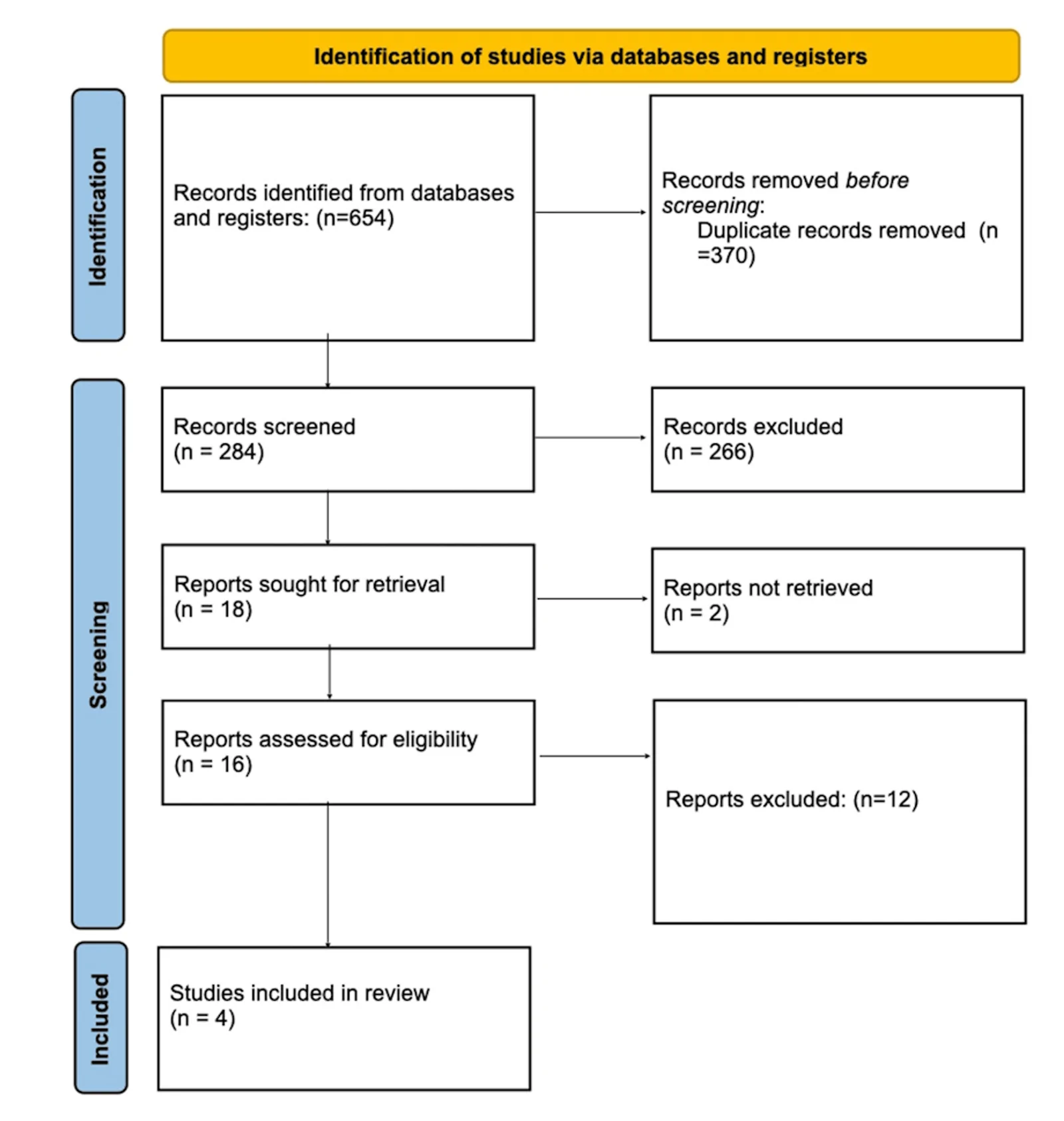
Study flow diagram.

A comprehensive search of several databases from each database's inception to July 14, 2026, was conducted. The databases included PubMed, Scopus, Embase, PsycINFO, Google Scholar, and Web of Science. We also searched ClinicalTrials.gov for ongoing trials. The search was designed using controlled vocabulary and keywords "autism spectrum disorder," "autistic disorder," "Asperger's," "PDD-NOS," and "valproate.” The search was performed in all languages and was limited to human subjects. The complete search strategy for PubMed is available in Appendix 1 (see Table 4). We also performed a manual search of references listed in the included studies to limit the risk of missing eligible studies (Figure 1). 

Study selection and eligibility criteria

Two reviewers (H and NG) independently screened the titles and abstracts of potentially eligible articles. Any disagreements during study screening or data extraction were resolved through discussion and agreement between the reviewers. If an agreement could not be reached, the senior author made the final decision. The study's inclusion criteria were: P (Population): Children with ASD defined by the Diagnostic and Statistical Manual of Mental Disorders (DSM-5/DSM-IV-TR) with the use of an Autism Diagnostic Observation Schedule (ADOS)/ADOS-2. I (Intervention): Studies evaluating divalproex sodium, valproic acid, or sodium valproate. C (Comparator): Treatment as usual including placebo, selective serotonin reuptake inhibitors (SSRI), and/or antipsychotics. O (Outcome): Primary outcomes included improvement in irritability, aggression, behavioral dysregulation, repetitive behaviors, and overall ASD-related symptoms. Secondary outcomes included hyperactivity, impulsivity, mood symptoms, and adverse effects. S (Study design): Clinical trials. Exclusion criteria were unpublished data.

Data collection

Data were collected from the included studies using a standardized data extraction form by two reviewers. Data were extracted on variables including study characteristics (author, year, country, study design, research question, sample size, demographic characteristics of study participants, inclusion, and exclusion criteria), intervention types, outcome measures, and duration of follow-up. No automated tools were used for screening, data extraction, or risk-of-bias assessment. All steps were completed manually by two independent reviewers.

Methodological quality and risk of bias assessment

**Figure 2:**
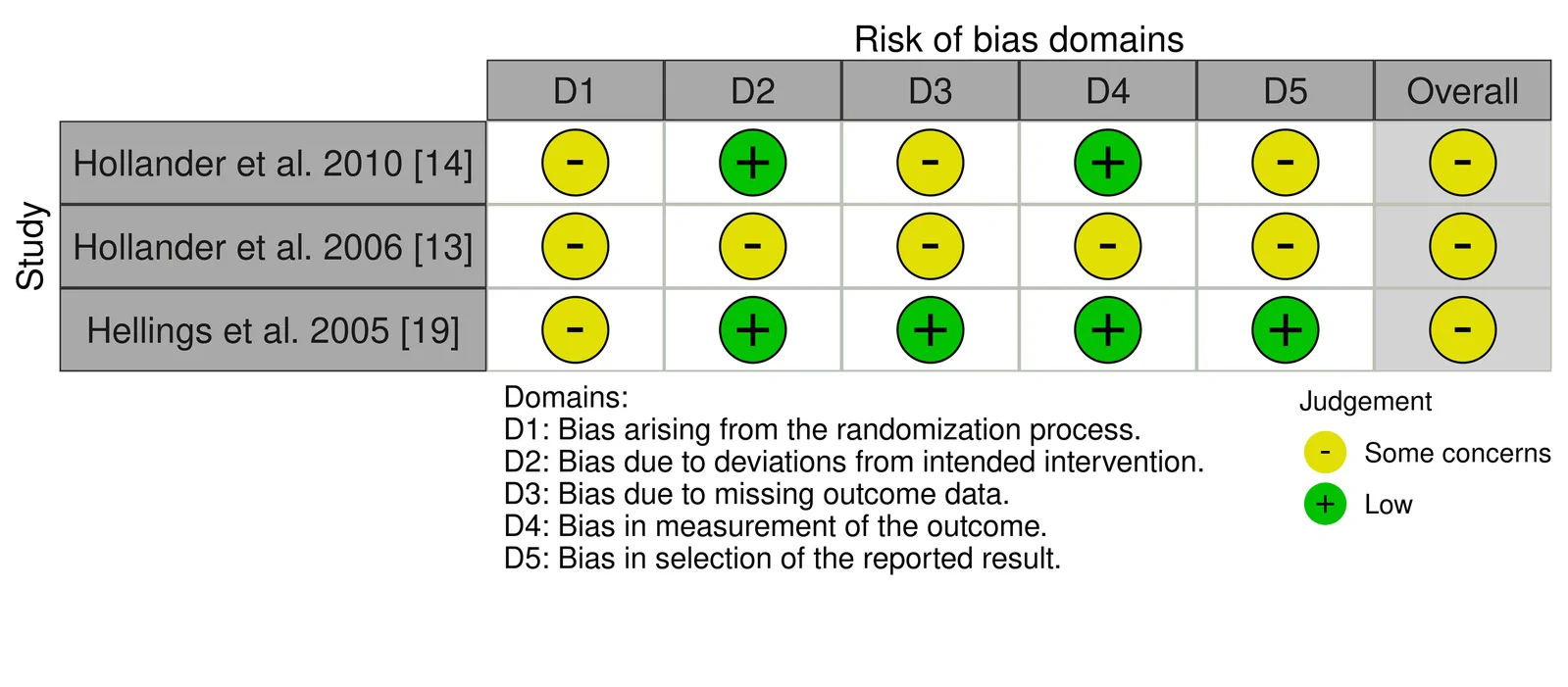
Risk of bias summary.

**Figure 3:**
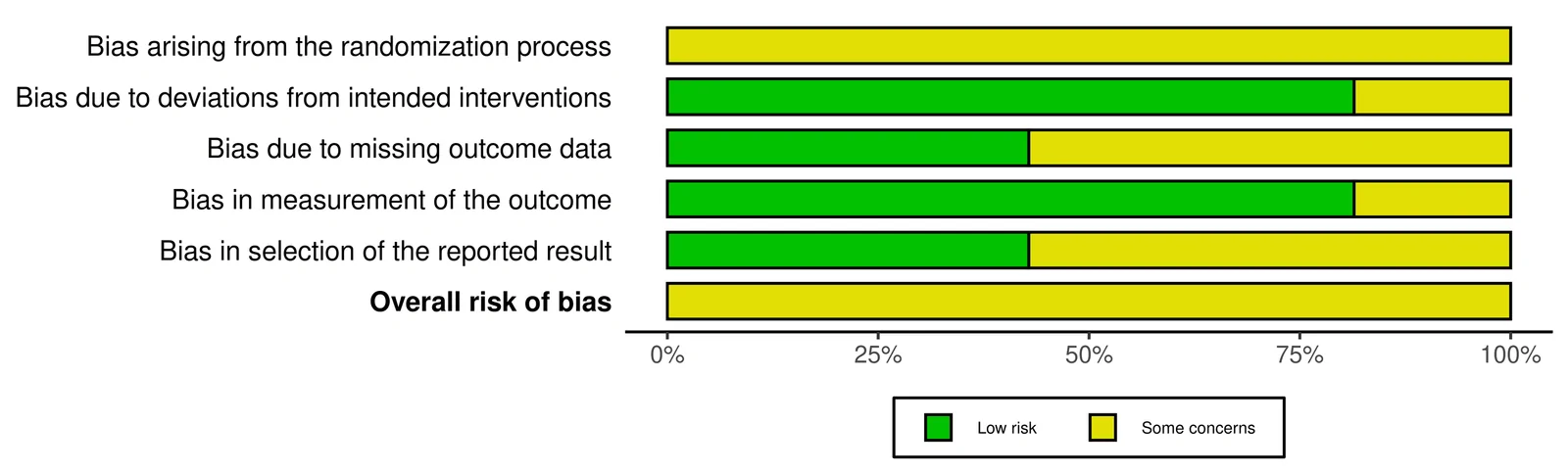
Risk of bias graph.

We used the revised Cochrane risk-of-bias tool for randomized trials (RoB 2) [11] to evaluate the methodological quality of the included randomized controlled trials (RCTs). Each trial was assessed on its primary efficacy outcome across five domains, including bias arising from the randomization process, bias due to deviations from intended interventions, bias due to missing outcome data, bias in measurement of the outcome, and bias in selection of the reported result, together with an overall risk-of-bias judgment (Figures 2, 3). Two reviewers independently assessed each trial and resolved disagreements through discussion. The open-label study was not included in this assessment, as RoB 2 applies only to randomized trials. ROBINS-I was also not used because it is designed for non-randomized studies that compare two or more groups, while this study had only one treatment group and no control group. Other tools for single-group studies were not used because the study has clear limitations, including no control group, no blinding, a small and mixed sample of participants, and the use of other psychiatric medications by most participants. Using these tools would not provide any additional useful information. Therefore, this study was assessed by describing its strengths and limitations, and its results should be considered preliminary and useful mainly for generating future research questions rather than proving that the treatment is effective. We did not use the Grading of Recommendations, Assessment, Development, and Evaluations (GRADE) approach because each outcome was supported by only one or two small studies that used different outcome measures, and no combined analysis was possible. A formal GRADE assessment would have resulted in very low certainty for all outcomes without providing meaningful differences between them.

Results

Study Characteristics 

The four studies on DVPX/VPA in ASD are heterogeneous, making it difficult to compare them. Ages ranged from 5 to 40 years, with two trials also enrolling adults [12,13]; diagnoses were autistic disorder, Asperger's disorder, and other pervasive developmental disorder-not otherwise specified (PDD-NOS). Nonverbal intelligence quotient (IQ) varied from a mean of about 63 in one trial to about 69 in another [12,14], and dosing was individualized by weight (10-20 mg/kg/day) or serum level (50-100 μg/mL) rather than fixed. Treatment also lasted eight to 12 weeks in every trial, too short to estimate lasting benefits or the metabolic and endocrine effects that may accumulate over months to years. Trials also used different outcome measures, including the Aberrant Behavior Checklist (ABC) Irritability subscale [15], the Clinical Global Impression scale [16], the Children's Yale-Brown Obsessive-Compulsive Scale [17], and the Overt Aggression Scale [18] in different combinations. That rules out including the data in a single quantitative estimate. It even makes qualitative comparison difficult. Sample sizes were small (13 to 30 participants), predominantly male, and from single centers; none of the trials sorted participants by symptoms, seizure or EEG status, or intellectual functioning, which is the kind of classification needed to separate responders from non-responders. Because the studies were so different, it is not possible to conclude DVPX/VPA efficacies. Instead, each study should be interpreted individually, considering its specific patient group and limitations. The reason we did not combine the study results was that the trials measured different outcomes using different assessment tools. Only two studies used the same primary outcome measure, and they reported opposite results. Differences in the study populations were another important reason, as participants ranged from 5 to 40 years of age and had different diagnoses. Study design also differed, with one study being open-label and having no control group. In comparison, the treatment methods were quite similar across the studies, as all used weight-based or blood level-guided dosing for 8 to 12 weeks (Table 1).

**Table 1:** Key clinical trials of valproate in autism spectrum disorder. ABC: Aberrant Behavior Checklist; ASD: autism spectrum disorder; CGI: Clinical Global Impression scale; CGI-I: Clinical Global Impression-Improvement scale; C-YBOCS: Children's Yale-Brown Obsessive Compulsive Scale; DVPX: divalproex; EEG: electroencephalography; PDD-NOS: pervasive developmental disorder-not otherwise specified; RCT: randomized controlled trial; VPA: valproate. Confidence intervals were not reported in the original publications. Effect sizes are given as reported by the study authors.

Study	Design and population	Outcome measure and result	Critical appraisal
Hollander et al., 2001 [12]	Open-label, N = 14 (autism, Asperger’s, PDD-NOS; with/without seizure history or EEG abnormality)	CGI-I; 10/14 (71%) rated as sustained responders, with the strongest signal in those with EEG abnormalities or seizure history	No control group; 10/14 on concurrent psychotropics; apparent benefit clusters in a seizure/EEG subgroup, raising the possibility of an antiepileptic rather than a primary behavioral effect
Hollander et al., 2010 [14]	RCT, N = 27, ages 5-17, 12 weeks	ABC-Irritability subscale and CGI-Improvement; 62.5% vs 9% responders on CGI-irritability (OR 16.7, Fisher's exact p = 0.008) and significant improvement on ABC-Irritability (p = 0.048); no improvement in core ASD symptoms	Allocation concealment not described; roughly 1 in 10 withdrew, some for lack of efficacy; modest sample limits power
Hollander et al., 2006 [13]	RCT, N = 13 (12 children/adolescents, 1 adult), 8 weeks	C-YBOCS; significant group difference in repetitive/compulsive behavior vs placebo (p = 0.037, d = 1.62)	Brief-report format; includes an adult participant; attrition and analytic approach not clearly described; authors characterize findings as preliminary
Hellings et al., 2005 [19]	RCT, N = 30, ages 6-20, 8 weeks	ABC-Irritability, CGI, Overt Aggression Scale; no significant difference vs placebo	High placebo response; single-center, heterogeneous, wide age range; underpowered to exclude a clinically meaningful effect

Evidence Synthesis

Epilepsy and EEG abnormalities: This is the condition where the use of DVPX/VPA is most strongly supported, since its anticonvulsant effects are well established on their own. Epilepsy affects a greater proportion of individuals with ASD than the general population, estimated roughly from 8% in ASD without intellectual disability to over 20% where intellectual disability is present, and epileptiform EEG abnormalities are found in up to 60% of children with ASD even without clinical seizures [20,21]. In the open-label trial by Hollander et al. [12], the subgroup with EEG abnormalities or a seizure history showed the most consistent benefit, with improvement in affective instability, impulsivity, and aggression alongside the expected anticonvulsant effect. Because most participants were taking other psychotropic medications and the study did not include a placebo group, it is unclear whether the observed benefits were due to DVPX/VPA's effects on ASD symptoms or its antiepileptic action. However, in children with ASD who also have epilepsy or an abnormal EEG, its use is already well established.

Irritability, aggression, and disruptive behavior: Here the evidence conflicts. Hollander et al. [14] randomized 27 children and adolescents to DVPX or placebo for 12 weeks and found a significant reduction in irritability and aggression on the ABC-Irritability subscale, with no improvement in social communication or restricted/repetitive behavior, which matches with its mechanism, that DVPX acts on behavioral reactivity, not core symptoms. Against this, Hellings et al. [19], in a comparable population of 30 individuals with pervasive developmental disorder and aggression, found no difference from placebo on the same ABC-Irritability measure, the Clinical Global Impression scale, or the Overt Aggression Scale, in a trial characterized by a high placebo response and single-center heterogeneous enrollment. Taken together, these two trials do not resolve the question. They show how easily the results shift with small changes in sample or design. Lithium and DVPX/VPA are considered less reliably effective than second-generation antipsychotics for irritability in ASD, and neither has the trial support that risperidone and aripiprazole have established [1].

Repetitive and compulsive behaviors: Hollander et al. [13] randomized 13 participants (12 children and adolescents and one 40-year-old adult) to DVPX or placebo for eight weeks, using the Children's Yale-Brown Obsessive-Compulsive Scale as the primary outcome, and reported significant improvement with DVPX. The authors themselves characterized the findings as preliminary. There are some reasons to be cautious. The study was published as a brief report, included one adult in a mainly pediatric sample, and did not provide detailed information about participant dropouts. Although it showed the largest effect, it was also the weakest of the four studies methodologically. Therefore, its findings should be interpreted with caution.

Mood Dysregulation, Bipolar Features, and Emotional Dysregulation

No ASD study has directly tested DVPX/VPA against placebo for mood problems or bipolar-like symptoms. Emotional dysregulation and mood instability overlap between ASD and bipolar disorder, sharing irritability, impulsivity, and affective lability as transdiagnostic features that are difficult to distinguish without a careful history [22]. Where ASD and bipolar disorder may co-occur, one secondary analysis found no difference in response rates among second-generation antipsychotics, which is the closest available evidence, but it is not specific to DVPX/VPA [1]. A recent meta-analysis of pharmacological interventions for irritability and emotional dysregulation in ASD found the largest effect sizes for aripiprazole and risperidone, and no significant benefit for mood stabilizers as a class over placebo [23]. In practice, the use of it for bipolar-spectrum features in ASD is based on evidence from adult and pediatric bipolar disorder, not from ASD-specific trials. This should be clearly explained to patients and families. 

Other Neuropsychiatric Comorbidities

A large percentage of children with ASD also have attention-deficit/hyperactivity disorder (ADHD), and their irritability is often chronic rather than episodic and frequently persists even after stimulants and alpha-agonists have been optimized. In that specific situation, adding risperidone or DVPX/VPA to stimulant treatment is supported by evidence from child psychiatry studies, including our previous review of off-label treatments in ASD [1]. This approach uses it as an add-on treatment. Overall, the evidence suggests that it is most useful for specific, well-defined co-occurring problems in ASD. The strength of evidence and the clinical takeaway for each phenotype are summarized in Table 2. 

Table 2 presents the strength of evidence for each clinical phenotype. Publication bias was not formally assessed because funnel plots and statistical tests for small-study effects are not useful with only four studies and no combined analysis.

**Table 2:** Evidence strength by clinical phenotype. Sources: The authors developed this synthesis using the mechanistic and clinical evidence discussed above, including Gupta and Gupta (2024) [1], the included clinical trials [12-14,19], and references [20-23]. EEG: electroencephalography; ASD: autism spectrum disorder; RCT: randomized controlled trial.

Phenotype	Basis for use	Strength of evidence	Clinical takeaway
Epilepsy/epileptiform EEG abnormality	Established anticonvulsant mechanism; subgroup signal in open-label data [12]	Strongest, though indirect (since valproate here treats a genuine comorbidity rather than a primary ASD trial-tested target) [20,21]	Standard anticonvulsant management; behavioral benefit a plausible secondary effect
Irritability/aggression	GABAnergic and antiglutamatergic dampening of behavioral reactivity	Mixed/modest (one positive, one negative RCT in comparable populations) [14,19]	Reasonable second-line option after antipsychotic failure or intolerance; not established as monotherapy of first choice
Repetitive/compulsive behavior	HDAC-mediated neuroplastic effects	Weak (one small, methodologically limited positive trial) [13]	Preliminary only; not sufficient to guide routine practice
Mood dysregulation/bipolar-spectrum features	Established mood-stabilizing mechanism in bipolar disorder [5,22]	Inferential (no ASD-specific placebo-controlled trial)	Extrapolated use; should be framed to families as such
Core social-communication/restricted-repetitive symptoms	No mechanistic prediction of benefit	Absent	Not an appropriate target for valproate

Discussion

Overall, the available evidence suggests that DVPX/VPA may be useful in selected cases of ASD with specific symptoms. The recommendations that follow represent an expert interpretation based on indirect evidence, mechanistic rationale, and clinical experience, and are not conclusions established by randomized ASD-specific trials.

Who is an Appropriate Candidate?

The strongest candidate is a child or adolescent with ASD who also has a documented seizure disorder or epileptiform EEG abnormality and prominent irritability, aggression, or affective instability. Here, DVPX/VPA treats a genuine comorbidity, and any behavioral improvement is a reasonable secondary benefit. A second possible group is children or adolescents with ongoing irritability or aggression who have tried risperidone or aripiprazole but did not improve or developed significant side effects. This approach is based on expert opinion rather than direct trial evidence, as none of the randomized DPX/VA trials included patients specifically selected for poor response or side effects from antipsychotics. Also, no included study directly compared DPX/VA with an antipsychotic medication. A third candidate is the individual with clear mood cycling or bipolar-spectrum features rather than chronic persistent irritability. This use is supported mainly by studies of adult mood disorders, but it has not been studied in people with ASD.

What Should be Tried First?

Before pharmacotherapy is initiated, behavior treatment, functional behavioral assessment, and parent training should already be in place. Medical and environmental contributors of irritability (for example: constipation, gastroesophageal reflux, dental pain, sleep disturbance, and sensory overload) should also be evaluated and addressed, since these are common, modifiable reasons linked to behavioral symptoms. Among pharmacologic options, risperidone and aripiprazole remain first-line given their FDA approval and larger effect sizes for irritability and should generally be tried at an adequate dose and for an adequate duration before DVPX/VPA is considered as monotherapy.

*What Justifies Moving to Divalproex Sodium*/*Valproate* *(DVPX/VPA)?*

Stepwise transition to DVPX/VPA is reasonable when first-line antipsychotics have failed, are not tolerated because of weight gain or extrapyramidal symptoms, or are being used at a dose the family and clinician wish to minimize because of metabolic concerns. In this situation, DVPX/VPA may be used along with an antipsychotic medication to help reduce the required antipsychotic dose. However, this idea is based on findings from other pediatric populations and has not been directly studied in ASD. None of the trials included in this review evaluated DVPX/VPA as an add-on treatment to antipsychotics or examined whether it could reduce antipsychotic doses. It is also appropriate for children with ASD who have a seizure disorder or epileptiform EEG, even if they have not tried antipsychotics, because it is a standard treatment for these conditions. Shared decisions should be made together with the family, with clear target symptoms and a planned review after 8 to 12 weeks. Treatment should be continued only if the benefits outweigh the risk.

Safety and Monitoring

DVPX/VPA carries a rare but serious risk of hepatotoxicity, which is common in young children, patients on multiple antiepileptic drugs, and those with an underlying metabolic or mitochondrial disorder [24]. Baseline and periodic liver function testing is necessary, though normal transaminases do not exclude the rare syndrome of hyperammonemic encephalopathy, which can occur without hepatic enzyme elevation and should be suspected in any patient who develops unexplained lethargy, vomiting, or altered mental status while on the drug [25,26]. Acute pancreatitis is a further rare but potentially life-threatening boxed-warning risk and should be suspected in any patient who develops new abdominal pain, persistent vomiting, or anorexia [24]. 

Mitochondrial dysfunction is more common in children with ASD than in the general pediatric population, and DVPX/VPA is specifically capable of causing fulminant liver failure in patients with underlying mitochondrial disease, including undiagnosed polymerase gamma (POLG)-related disorders [27,28]. A careful history for developmental regression, unexplained hypotonia, or a family history of mitochondrial or metabolic disease is necessary before starting DVPX/VPA, and the threshold for genetic evaluation should be low in any child with an atypical or severe presentation.

Thrombocytopenia is a rare adverse effect at higher DVPX/VPA levels, generally above 110 to 135 μg/mL, and depends on both dose and serum concentration. Periodic complete blood counts are required, especially during dose titration or before procedures [25,26].

Weight gain is a well-documented long-term effect and should be monitored alongside body-mass index, particularly since many individuals with ASD are already at elevated risk of antipsychotic-associated weight gain from prior or concurrent treatment. In women and girls of reproductive age, DVPX/VPA is associated with an increased risk of polycystic ovary syndrome (PCOS), hyperandrogenism, and menstrual irregularity, effects that can appear within months of starting treatment and are only partially reversible on discontinuation [29]. This risk, together with VPA's teratogenicity, makes it inappropriate in this cohort unless alternatives have been exhausted.

DVPX/VPA carries one of the highest teratogenic risks out of any commonly used psychotropic or anticonvulsant, with a roughly ten- to twenty-fold increase in neural tube defects attributed in part to interference with folate metabolism and to epigenetic and oxidative mechanisms operating during early organogenesis [30]. It should be avoided in girls and women of childbearing potential unless no acceptable alternative exists, and its use in this population requires effective contraception and documented counseling about reproductive risk, ideally revised at every visit rather than only at initiation [31]. Prenatal exposure to DVPX/VPA is widely used to create a rodent model of ASD because it consistently produces autism-like behaviors in offspring [10].

DVPX/VPA inhibits glucuronidation and can roughly double serum lamotrigine concentrations when the two are combined, raising the risk of Stevens-Johnson syndrome. Combination therapy requires a slower lamotrigine titration and closer dermatologic monitoring [32]. Its hepatic enzyme effects also need caution with other hepatically metabolized agents and with other antiepileptic drugs used in polytherapy.

The studies lasted only 8 to 12 weeks, which is too short to detect long-term side effects such as weight gain, hair loss, tremor, sedation, and hormonal changes. They also did not assess bone health, daily functioning, or quality of life with long-term use. Therefore, patients receiving long-term DVPX/VPA should be monitored regularly, as good short-term tolerance does not mean long-term safety.

Knowledge Gaps and Future Research Priorities

A major limitation of the current evidence is that none of the four studies identified which groups of patients are most likely to benefit. The studies did not group participants by seizure or EEG status, symptom type, or biological markers, even though the available evidence suggests that some subgroups may respond better than others. Future studies should compare groups such as those with and without EEG abnormalities, those with mainly irritability versus repetitive behaviors, and those with or without bipolar-spectrum features, instead of treating all individuals with ASD as one group. As a future research area, studies should examine whether biomarkers such as quantitative EEG, genetic factors related to medication tolerance, and serum DVPX/VPA levels can help identify which patients may benefit from treatment. However, none of these approaches are currently supported by evidence from this review, and they should not be used to guide treatment decisions at this time. Larger sample sizes and longer randomized controlled trial durations are also needed, both with DVPX/VPA alone and in combination with antipsychotics, using standardized outcome measures to allow comparison across studies. Long-term safety data are also lacking because no study has followed growth, bone health, hormonal function, or daily functioning over extended periods. Finally, studies comparing DVPX/VPA directly with approved antipsychotics and behavioral therapy would help define its role in the treatment of ASD.

Practical considerations for candidate selection, risk mitigation, and monitoring during DVPX/VPA use in pediatric ASD are summarized in Table 3.

**Table 3:** Practical considerations for valproate use in pediatric ASD. Sources: Authors' recommendations synthesized from the safety and monitoring literature cited in this review, including references [24-32] and the U.S. product labelling [25]. EEG: electroencephalography; ASD: autism spectrum disorder; PCOS: polycystic ovary syndrome; POLG: polymerase gamma.

Consideration	Recommendation
Clinical phenotype most likely to benefit	Children/adolescents with prominent irritability, aggression, or behavioral dysregulation, repetitive/compulsive behavior, coexisting mood instability, or comorbid seizures/EEG abnormalities; unlikely to improve core social-communication deficits [12-14,19]
Phenotypes best avoided	Girls and women of childbearing potential (unless alternatives fail), known or suspected mitochondrial/metabolic disease including POLG-related disorders, children under 2 years, antiepileptic polytherapy, hepatic impairment [26,27,29,30]
Risk mitigation	Lowest effective dose titrated to serum level; effective contraception and documented counselling in females of reproductive age; screen for mitochondrial disease before starting; reduce and slow-titrate lamotrigine if combined; consider carnitine in high-risk children [30,31]
Monitoring strategy	Baseline complete blood count (CBC), liver function test (LFT), weight/body mass index (± pregnancy test); periodic LFTs, platelet count, and valproate level; check ammonia with unexplained lethargy or mental-status change [24-26]
Long-term problems to watch	Weight gain and metabolic change, tremor, sedation, hair loss, menstrual irregularity/PCOS features and other endocrine effects, dose-related thrombocytopenia, rare hepatotoxicity, possible effects on bone health, ongoing teratogenic risk through the reproductive years [29,30]

Strengths and Limitations

The strengths of this review include a pre-registered protocol, a search of six databases and a clinical trial registry without language restrictions, independent duplicate screening and data extraction, formal assessment of risk of bias in randomized trials, and organization of the evidence according to clinical symptoms relevant to treatment decisions.

This review is limited by the poor evidence base. Only four studies were identified, and three had small sample sizes, making it difficult to draw conclusions. Three of the four included trials were conducted by the same research group, and at least one study received funding from the manufacturer of DVPX. Therefore, the available evidence is not fully independent and may have potential commercial influence. Formal risk-of-bias assessment was also limited to the three randomized trials, with the open-label trial appraised narratively for the reasons given in the Methods. Although our focus was on children and adolescents, some included trials also enrolled adults and did not stratify outcomes by age, and diagnostic criteria varied across studies, limiting the consistency of the population. Our phenotype-based approach is based on indirect evidence, mechanistic rationale, and clinical judgment, and goes beyond what was directly tested in the included trials. We did not perform a meta-analysis because the studies were too different to combine. Instead, our conclusions are based on a qualitative review of the evidence. As with any review, our interpretation may be influenced by the authors' clinical judgment, although we have attempted to make our reasoning clear throughout.

## Conclusions

DVPX/VPA has a plausible rationale in ASD through its GABAergic, antiglutamatergic, and histone deacetylase-mediated actions and weak clinical evidence based on four small trials, inconsistent findings for irritability and aggression, one preliminary positive trial for repetitive behavior, and essentially no direct evidence for mood dysregulation or bipolar-spectrum presentations beyond extrapolation from other studies. The most compelling use is in children with comorbid seizures or epileptiform EEG abnormalities, where DVPX/VPA is standard anticonvulsant care with a plausible behavioral co-benefit, and in patients with persistent irritability who have not responded to first-line antipsychotics. Given its teratogenic, hepatic, hematologic, and endocrine risks, valproate is not a routine or first-line choice for ASD-associated behavioral symptoms, and its use should rest on a clearly defined target symptom, informed family counseling, and structured monitoring rather than the assumption of its traditional mood stabilizer effects.

## Appendices

Appendix 1 

**Table 4:** Complete PubMed search strategy. Search run from database inception to July 14, 2026. No language, publication-date, or study-design restrictions were applied. MeSH: Medical Subject Headings; tiab: title/abstract field tag; *: truncation operator; quotation marks denote exact-phrase searching. Line #8 applies the standard PubMed animal-exclusion algorithm, removing records indexed as Animals without a corresponding Humans index term. The same two concept blocks were adapted to the controlled vocabulary and field syntax of each remaining database.

Line	Search query	Records
#1	"Autism Spectrum Disorder"[MeSH] OR "Autistic Disorder"[MeSH] OR "Asperger Syndrome"[MeSH] OR "Child Development Disorders, Pervasive"[MeSH]	
#2	autis*[tiab] OR Asperger*[tiab] OR "PDD-NOS"[tiab] OR "pervasive developmental disorder"[tiab] OR "pervasive developmental disorders"[tiab]	
#3	#1 OR #2	
#4	"Valproic Acid"[MeSH]	
#5	valpro*[tiab] OR divalproex*[tiab] OR Depakote[tiab] OR Depakene[tiab] OR Depakine[tiab] OR Epilim[tiab] OR Convulex[tiab] OR Orfiril[tiab] OR Epival[tiab]	
#6	#4 OR #5	
#7	#3 AND #6	
#8	#7 NOT ("Animals"[MeSH] NOT "Humans"[MeSH])	430
